# Study Protocol of European Regulatory Science on Tobacco (EUREST-PLUS): Policy implementation to reduce lung disease

**DOI:** 10.18332/tid/93305

**Published:** 2018-08-21

**Authors:** Constantine I. Vardavas, Nicolas Bécuwe, Tibor Demjén, Esteve Fernández, Ann McNeill, Ute Mons, Yannis Tountas, Antigona C. Trofor, Aristides Tsatsakis, Gernot Rohde, Marc Willemsen, Krzysztof Przewozniak, Witold A. Zatonski, Geoffrey T. Fong, on behalf of the EUREST-PLUS consortium

**Affiliations:** 1 European Network on Smoking and Tobacco Prevention (ENSP), Brussels, Belgium; 2 University of Crete (UoC), Heraklion, Greece; 3 Kantar Public (TNS), Brussels, Belgium; 4 Smoking or Health Hungarian Foundation (SHHF), Budapest, Hungary; 5 Institut Català d’Oncologia (ICO) and Bellvitge Biomedical Research Institute (IDIBELL), Catalonia, Spain; 6 School of Medicine and Health Sciences, Universitat de Barcelona, Catalonia, Spain; 7 King’s College London (KCL), London, United Kingdom; 8 Cancer Prevention Unit and WHO Collaborating Centre for Tobacco Control, German Cancer Research Center (DKFZ), Heidelberg, Germany; 9 University of Athens (UoA), Athens, Greece; 10 University of Medicine and Pharmacy ‘Grigore T. Popa’, Iasi, Romania; 11 Aer Pur Romania, Bucharest, Romania; 12 European Respiratory Society (ERS), Lausanne, Switzerland; 13 School CAPHRI, University of Maastricht (UniMaas), Maastricht, The Netherlands; 14 Health Promotion Foundation (HPF), Warsaw, Poland; 15 University of Waterloo (UW), Waterloo, Canada; 16 Ontario Institute for Cancer Research, Toronto, Canada

**Keywords:** tobacco control, WHO FCTC, tobacco products directive, policy implementation, regulatory science

## Abstract

Efforts to mitigate the devastation of tobacco-attributable morbidity and mortality in the European Union (EU) are founded on its newly adopted Tobacco Products Directive (TPD) along with the first-ever health treaty, the WHO Framework Convention on Tobacco Control (FCTC). The aim of this Horizon 2020 Project entitled **European Regulatory Science on Tobacco: Policy Implementation to Reduce Lung Disease (EURESTPLUS)** is to monitor and evaluate the impact of the implementation of the TPD across the EU, within the context of WHO FCTC ratification. To address this aim, EUREST-PLUS consists of four objectives: 1) To create a cohort study of 6000 adult smokers in six EU MS (Germany, Greece, Hungary, Poland, Romania, Spain) within a pre-TID vs post-TPD implementation study design; 2) To conduct secondary dataset analyses of the Special Eurobarometer on Tobacco Survey (SETS); 3) To document changes in e-cigarette product parameters (technical design, labelling/packaging and chemical composition) pre-TID vs post-TPD; and 4) To enhance innovative joint research collaborations on chronic non-communicable diseases. Through this methodological approach, EUREST-PLUS is designed to generate strong inferences about the effectiveness of tobacco control policies, as well as to elucidate the mechanisms and factors by which policy implementation translates to population impact. Findings from EUREST-PLUS have potential global implications for the implementation of innovative tobacco control policies and its impact on the prevention of lung diseases.

## INTRODUCTION

Tackling tobacco consumption is essential to reducing the detrimental impact of chronic non-communicable diseases. Tobacco use is considered as the single most significant cause of preventable morbidity and mortality in the European Union (EU) and worldwide^1^. In the EU, tobacco use is responsible for over 650000 premature deaths annually, half of which are among those who are 35–69 years old^2,3^. In addition to the pervasive effect on public health, tobacco-related death and illness have severe implications for the efficiency and cost-effectiveness of health care delivery systems, exacerbating the economic recession of many European Union Member States (EU MS) that are already under severe financial constraints^4,5^. Efforts to mitigate the devastation of tobacco-attributable morbidity and mortality in the EU consist of its newly adopted Tobacco Products Directive (TPD)^6^ and the first-ever health treaty, the WHO Framework Convention on Tobacco Control (FCTC)^7^.

The WHO FCTC is a comprehensive framework treaty that has propelled national and international tobacco control efforts forward, by setting forth a comprehensive legal framework to guide Parties in integrating the recommended policies and measures into their own national legislation. Such policies include increased taxes on tobacco products, comprehensive smoke-free laws, limitations or bans on tobacco advertising, promotion and sponsorship, and support for tobacco cessation services, amongst other measures. The TPD on the other hand regulates primarily the tobacco product itself, including its ingredients, additives, packaging, labelling, reporting and aspects related to novel tobacco products and e-cigarettes (European Comission, 2014). Within Europe and after substantial debate, the new TPD replaced the previous TPD, (implemented in 2011 under Directive 2001/37/EC) and was finally adopted by the Council and the European Parliament on 29 April 2014 with its Articles that were to be implemented by 22 May 2016 across the EU MS. (European Comission, 2014). However, in order to maximize TPD’s impact and to keep abreast with the ever-changing tobacco and nicotine sector, it is expected that the foundational elements of the TPD will be iteratively shaped by the emergence of new scientific evidence, so that the Acts of the TPD can be fully operationalized. Assessment reports have already given significant shape to the regulation of reporting requirements across the EU, the identification and banning of characterising flavours in tobacco products, and the regulation of refill mechanisms of e-cigarettes^8-10^. These two instruments, the WHO-FCTC and the TPD, in tandem, provide a comprehensive framework of actions that, if appropriately adopted and subsequently implemented by EU MS, would have the potential to greatly reduce tobacco use initiation, increase cessation and reduce demand for tobacco products.

In light of the above regulatory changes, the mission of EUREST-PLUS is to monitor and evaluate the impact of the implementation of the TPD across the EU, within the context of WHO FCTC ratification. To address its mission, EUREST-PLUS established four core objectives, which are fulfilled through seven integrated work packages (Table 1), five being research and innovation oriented (vertical) and two support oriented (horizontal), over the course of 36 months.

**Table 1:** An overview of EUREST-PLUS Work Packages (WPs)

*EUREST-PLUS Work Packages*
**WP1:** Coordination and Management
**WP2:** Cohort Study on Tobacco Control in the EU: Wave 1 (ITC Project)
**WP3:** Cohort Study on Tobacco Control in the EU: Wave 2 (ITC Project)
**WP4:** Cross-country analysis among ITC cohorts: EU and non-EU pooled analyses
**WP5:** Secondary analyses of the Special Eurobarometer on Tobacco Surveys
**WP6:** Assessing e-cigarette product compliance to the TPD
**WP7:** Maximising impact through communication and dissemination

## METHODS

The background and methodological approach for each of the four linked core objectives of the EUREST-PLUS Project are described below.

### Objective 1: To evaluate the psychosocial and behavioural impact of the implementation of the TPD Articles and policies outlined in the WHO FCTC, through the creation of a cohort of 6000 adult smokers in six EU MS (Germany, Greece, Hungary, Poland, Romania, Spain) within a pre-TPD vs post-TPD implementation study design (WP2, WP3)

The conceptual model of this objective of EURESTPLUS is based on the ITC Project’s theory-driven framework that hypothesizes the pathways of tobacco control policies on tobacco use behaviours^11^. Rooted in psychosocial theories of health behaviour, including the Health Belief Model^12^, Social Cognitive Theory^13^, Theory of Planned Behavior^14^, and Protection Motivation Theory^15^, these models serve as an organizing principle for the content of the ITC surveys, research hypotheses, and data analysis^11^. Through the EUREST-PLUS Project, the ITC Project was expanded to include five EU MS (Greece, Spain, Romania, Poland, Hungary) and the reactivation of one EU MS (Germany) through a pre-TPD recruitment survey (Wave 1) and a post-TPD implementation follow up survey (Wave 2). These six EU MS were selected to represent the broad geographic and economic diversity of the EU. The central characteristics of these selected countries are presented in Table 2. The ITC cohorts in these six countries join existing ITC cohorts in the Netherlands, France and UK, creating opportunities for the evaluation of the TPD across nine EU MS.

**Table 2:** Characteristics of the participating EU MS in EUREST-PLUS

*Country*	*Geography*	*Volume indices of GDP per capita compared to EU-28 average, 2015 (33)*	*Unemployment Rate, 2015 (34)*	*Current Tobacco Use Prevalence, 2015§ (1)*	*Tobacco Control Scale Ranking 2016 (Ranking 2013) (35,36)*
Germany	West EU	24%	above 4.6%	30.9%	33 (33)
Greece	South EU	32% below	24.9%	43.3%	31 (29)
Spain	South EU	10% below	22%	29.7%	8 (7)
Poland	East EU	31% below	7.5%	28.6%	15 (20)
Hungary	East EU	32% below	6.8%	31.1%	9 (11)
Romania	East EU	43% below	6.8%	30.1%	7 (19)
France*	West EU	7% above	10.4%	32.8%	4 (5)
United Kingdom*	North EU	8% above	5.4%	23.1%	1 (1)
The Netherlands*	West EU	28% above	6.9%	26.4%	9 (13)

* Comparison participant (existing cohort)

§ Age-standardized prevalence estimates for current tobacco smoking among persons aged 15 and older, 2015

A cohort of 1000 adult (≥18 years old) smokers were recruited by multistage cluster sampling in each of 6 EU MS, resulting in a sample of smokers that is representative of each country (in total 6000 adult smokers). Sampling frames in each country were created through a national probability sampling design, comprising a geographic sample stratification based on Nomenclature of Territorial Units for Statistics (NUTS). After obtaining ethical approval from all participating centers and after the provision of informed consent, participants were asked to respond to a comprehensive survey that consisted of questions aimed to measure intermediary effect indicators of the introduction of TID provisions (e.g. health warnings, additives, e-cigarettes) and WHO FCTC Articles on demand reduction (e.g. smoke-free laws, tax/price policies, smoking cessation). The ITC Project has developed and validated key indicators of the effectiveness of each FCTC policy domains^16^. Also included in the ITC surveys are extensive sets of theory-driven psychosocial mediators (e.g. beliefs and attitudes, perceived risk, intentions to quit) and moderators (e.g. sociodemographics, respiratory comorbidities), as outlined in Table 3, which provide valuable measures of known precursors of future behaviour change important to public health (such as quit attempts and short- and long-term quit attempt success).

**Table 3:** EUREST-PLUS objectives in relation to work packages (WPs), WHO FCTC and TPD Articles, and participating countries

*Objective*	*WPs*	*WHO FCTC Articles*	*TPD Articles*	*Countries*
1) To evaluate the`psychosocial and behavioural impact of TPD and WHO FCTC implementation, through the creation of a cohort of 6000 adult smokers across 6 EU MS	WP2 WP3	Price and tax measures (6) Second-hand smoke (SHS) legislation (8) Product ingredients (10) Product labelling (11) Public awareness activities (12) Tobacco advertising, promotion and sponsorship (13) Illicit trade (15) Smoking cessation (14)	Ingredient Reporting (5) Additive Reporting (6) Characterising flavour (7) Product labelling (9–12) Packaging and presentation (13–14) Package traceability (15) Cross border sales (18) E-cigarettes (20)	6 EU MS (Germany, Greece, Hungary, Poland, Romania, Spain)
2) To assess support for TPD implementation and to monitor progress in WHO FCTC implementation through secondary dataset trend analyses of the Special Eurobarometer on Tobacco Survey (SETS)	WP5	Price and tax measures (6) SHS legislation (8) Tobacco advertising, promotion and sponsorship (13) Smoking cessation (14) Tobacco packaging and labelling (9)	Tobacco packaging and labelling (8–14) Tobacco product design characteristics (6, 7, 13) E-cigarettes (20)	All 28 EU MS
3) To document changes in e-cigarette product parameters (technical design, labelling/ packaging and chemical composition) following WHO TPD implementation.	WP6		E-cigarettes (20)	6 EU MS, European ITC Project countries (France, UK, Netherlands)
4) To enhance innovative joint research collaborations on chronic, non-communicable diseases (NCDs) in lowand middle-income countries (LMICs) and in vulnerable populations in high-income countries (HICs).	WP4 WP7	Demand reduction Articles Cooperation and communication (20–22)		All 28 EU MS and non-EU LMICs and HICs (Australia, Canada, Kenya, Thailand, United States, Uruguay, Zambia)

### Objective 2: To assess support and impact of TPD implementation and progress in WHO FCTC implementation through secondary dataset analyses of the Special Eurobarometer on Tobacco Survey (SETS)

The Special Eurobarometer on Tobacco Surveys (SETS) is a public health surveillance tool that aims to identify current consumption of tobacco products, examine perceptions and behaviours of tobacco users, and ultimately inform measures to reduce the burden of tobacco use in the EU^17^. SETS is a repeated cross-sectional survey of adults (≥15 years old) in all 28 EU MS, performed through in-person interviews at participants’ homes in their respective native language using a multi-stage sample design.

WP5 of EUREST-PLUS was designed to maximize the evidence that can be obtained from the vast repository of data available by conducting secondary data analyses that examine the relationship between individual-level tobacco-related measures and specific FCTC and TPD policy Articles. These analyses focus on the associations between sociodemographic and personal parameters with specific FCTC and TPD policy determinants that include, but not limited to: 1) Price and tax measures; 2) Protection from exposure to SHS; 3) Tobacco advertising, promotion and sponsorship; 4) Tobacco dependence and cessation; 5) Tobacco packaging and labelling; 6) Tobacco product design characteristics; and 7) E-cigarettes as outlined in Table 3. Within this WP, logistic regression analyses are performed to assess correlates of selected variables related to TPD preparedness and WHO FCTC implementation. The models are fitted for age, EU region, scope of national tobacco control policies, education, SES, gender and area of residence. Analyses stratified for young age (18–24 years), unemployment status, self-placement on the social ladder and the ability to pay bills are used as proxies to identify vulnerable populations (18–28 years).

### Objective 3: To document changes in e-cigarette product parameters (technical design, labelling/ packaging and chemical composition) following TPD implementation of TPD Art. 20 (WP6)

With a rapidly increasing and evolving market of diverse e-cigarette products and varying approaches to their regulation among EU MS, the TPD sets forth standardized safety and quality requirements for e-cigarette products. Article 20 of the TPD mandates that e-cigarette products comply with specifications regarding their design features, chemical composition, safety and labelling practices, amongst others. According to Art. 20(13) TPD, *the Commission shall, ‘lay down (…) technical standards for the refill mechanism provided for in paragraph 3(g) of Art. 20’* (European Comission, 2014). WP6 assesses implementation of Article 20, in regard to such use of technical standards and design characteristics of e-cigarette products, according to the specific parameters set forth by the TPD.

Within this WP, randomly selected samples from the products of the most popular e-cigarette brands were identified through sales data in 9 EU MS participating in EUREST-PLUS (Greece, Germany, UK, France, Poland, Romania, Spain, Netherlands, Hungary), both before and after Article 20 of the TPD was implemented across the EU, so as to evaluate parameters that include: a) *Labelling/packaging practices* (including labelling information and warnings that may be either within or on the packaging of the products); b) *Technical design/safety features* (emphasis on design features, including the existence of child resistant caps, tamper proof design, and the refill mechanism [TPD, Art. 20, p2e]); and c) *Chemical composition* (Analyses include the products nicotine content, flavours, humectant chemical composition, while a qualitative and quantitative evaluation, where possible, of all compounds in the samples is also performed)^29^.

### Objective 4: To enhance innovative joint research collaborations on chronic non-communicable diseases (NCDs) in LMICs and in vulnerable populations in HICs (WP4, WP7)

Given the lack of feasibility to conduct randomised controlled trials of policies implemented at the population level, cross-country comparisons are critical to health policy analyses as they enable, for instance, the comparison of tobacco use and quitting behaviours in populations exposed to the implementation of a policy compared to those that are not. Additionally, cross-country comparisons can provide valuable information about whether tobacco control policy impact varies by disparities or vulnerability, as well as within countries. Within EUREST-PLUS cross-sectional and longitudinal cross-country comparisons are performed. These enable the examination of differences and similarities between MS before the implementation of the EU TPD, and longitudinal comparisons of any cross-country differences in *changes* after the implementation of the EU TPD. The Wave 1 and Wave 2 cross-sectional and longitudinal data are also compared with: 1) data collected from the 6 EU MS in this project (Germany, Greece, Hungary, Poland, Romania, and Spain); 2) data collected from other EU MS involved in the ITC Project (UK, France, Netherlands); and 3) data collected from selected non-EU counties from the ITC Project (e.g. Australia, Canada, Kenya, Thailand, United States, Uruguay, Zambia), many of which are LMICs.

The objectives of the cross-country comparisons are prioritised around the evaluation of the implementation and impact of: 1) the EU TPD, e.g. new larger pictorial health warnings; and 2) the demand-related Articles of the WHO FCTC, e.g. smoke-free laws. The cross-country comparisons with EU countries focus on whether there are differences or commonalities in the impact of the EU TPD and the WHO FCTC policies, and whether there are any differences in implementation and enforcement of the EU TPD and WHO FCTC policies across countries that may explain any differences in impact.

## DISCUSSION

The EU’s TPD is a unique preventive action because of the demographic weight of the EU (511 million inhabitants), the fact that MS have to transpose the directive’s legal requirements directly into national law and the impact EU directives may have on non-EU countries. This legislative action also serves as a unique natural experiment of the effect of tobacco regulation on population outcomes across the 28 EU MS, and it is this unprecedented window of opportunity in which the EUREST-PLUS project was conceived.

Policy implementation and its subsequent population impact are dependent on the balance between barriers and facilitators, within the context of implementation into national legislation. EURESTPLUS is designed to generate strong inferences about the effectiveness of population-level policies. Hence, EUREST-PLUS will not only elucidate the mechanisms of policy impact, but will also explain how the impact of policies may be negatively influenced by challenges at a national level, such as high national smoking prevalence, low adherence to previous policies, population groups that have been difficult to reach by past programs and interventions, including low SES/equity groups, or challenges at the individual level (such as positive perceptions, attitudes and beliefs about smoking, psychosocial contexts that inhibit quitting among smokers, low self-efficacy to quit, and low intentions to quit). EUREST-PLUS may also have a significant impact on the prevention of lung diseases in Europe as it can provide the evidence needed by regulators to fuel tobacco control policies. As smoking is a major cause of lung diseases, any decrease in smoking and its immediate precursors (e.g. increase in quit attempts) can be interpreted as key indicators of subsequent smoking rates and the associated risk of lung diseases. Thus, the measurement of the impact of tobacco control measures on these precursors of downstream behaviour is critically important in linking the interventions to downstream health outcomes and prevalence of lung diseases. The impact of the project on reducing lung disease would be measured indirectly, through its effect on tobacco use and perceptions towards tobacco policy. Furthermore, the EUREST-PLUS Project is strengthened by its evaluation framework of the ITC Project, which through its rigorous methodology allows for a large representative cohort of the EU nations, which can also be compared to other parallel ITC cohorts on several tobacco use and control domains globally^11^. This framework is a key strategy not only to the core functions of public health-assessment, policy development and assurance^30^, but also in the implementation of the WHO FCTC through its strong alignment with the ‘M’ of the WHO MPOWER framework to monitor tobacco use and prevention policies^31^, a key tobacco control priority area for the WHO Europe Region^31^.

The present study design framework has some limitations, particularly in that self-reported survey data may be subjected to response bias, although the rigorous nature of the prospective cohort design allows for some minimization of such bias. A comprehensive understanding of the impact of tobacco control policies is also challenging without the inclusion of non-smokers in the study population. Further, the cohort of the EU MS in the current project may not be representative of all 28 EU MS, due to the vast social and cultural variability of populations across the EU. These limitations notwithstanding, the EUREST-PLUS project is a significant launching pad on which future research can be built upon for the continued monitoring and evaluation of the impact of tobacco control policies, both within the contexts of the WHO FCTC and EU TPD.

## CONCLUSIONS

The strong methodological approach and the timing of EUREST-PLUS are central to its innovative potential on a global scale, as the implementation of the EU TPD is a unique opportunity both to evaluate the effectiveness of new tobacco control policies adopted in the EU and to transfer this knowledge to other areas of the globe. At this historical moment in EU public health policy, the EUREST-PLUS project will be able to provide solid evidence that can fuel tobacco control, thus serving as a catalyst for future action in tobacco regulation.
